# Hemoglobin Trajectories on SGLT2 Inhibitor Therapy in Heart Failure: Anemia Marks Adverse Prognosis While Erythrocytosis Is Transient and Not Associated with Adverse Outcomes

**DOI:** 10.3390/jcm15145465

**Published:** 2026-07-13

**Authors:** Ivana Jurin, Irzal Hadžibegović, Marin Pavlov, Matea Marović, Tomislav Čikara, Marko Lucijanić

**Affiliations:** 1 Department of Cardiovascular Diseases, University Hospital Dubrava, 10000 Zagreb, Croatia; ivanajurin1912@gmail.com (I.J.); marin.pavlov@gmail.com (M.P.);; 2 Faculty of Dental Medicine and Health Care, Josip Juraj Strossmayer University of Osijek, 31000 Osijek, Croatia; 3 School of Medicine, University of Zagreb, Šalata 3, 10000 Zagreb, Croatia; 4 Division of Hematology, University Hospital Dubrava, 10000 Zagreb, Croatia; 5 Scientific Research and Translational Medicine Department, University Hospital Dubrava, 10000 Zagreb, Croatia

**Keywords:** heart failure, dapagliflozin, empagliflozin, hemoglobin, anemia, erythrocytosis

## Abstract

**Background/Objectives:** Sodium-glucose cotransporter 2 inhibitors (SGLT2is) raise hemoglobin in patients with heart failure (HF). We characterized the prevalence, dynamics, predictors and prognostic implications of three hemoglobin states (anemia, normal, and erythrocytosis) at baseline and during the first year on SGLT2i therapy. **Methods:** Prospective single-center registry of 1244 HF patients with a baseline hemoglobin who initiated dapagliflozin or empagliflozin (March 2022–October 2025). Hemoglobin was categorized at baseline, 6 and 12 months using WHO sex-specific thresholds. We analyzed inter-category transitions, predictors of each hemoglobin category at each timepoint, time-to-event outcomes (all-cause death, MACE, thrombosis) in landmark analyses, and the correlation of hemoglobin change with concurrent changes in left ventricular ejection fraction and NT-proBNP. **Results:** At baseline, 23.6% had anemia, 71.1% were normal, and 5.3% had erythrocytosis. The prevalence of erythrocytosis remained low throughout, with a modest, transient rise to 8.0% at 6 months before returning toward baseline (5.9% at 12 months), even though the underlying composition of patients was highly dynamic. Among baseline-normal patients, downward transition to anemia (7.8% at 6 months, 9.2% at 12 months) occurred at rates equal to or greater than upward transition to erythrocytosis (7.7% and 6.0%, respectively). The prevalence of anemia decreased significantly between baseline and the follow-up timepoints. Across timepoints, the patient’s own baseline hemoglobin was the dominant independent predictor of both subsequent erythrocytosis and subsequent anemia. Other independent predictors of erythrocytosis were higher adherence to SGLT2i, male sex, current smoking, COPD or asthma, higher heart rate and lower serum albumin. Other independent predictors of anemia were non-adherence to or discontinuation of SGLT2i, peripheral arterial disease, advanced age, diabetes, lower eGFR, higher NT-proBNP and lower serum albumin. Increases in hemoglobin from baseline were associated with greater concurrent reductions in NT-proBNP at 6 and 12 months but were not associated with changes in LVEF at either timepoint. Anemia was independently associated with all-cause death at the baseline and 6-month landmarks (adjusted HRs are 2.23 and 2.45, respectively; time-varying HR is 2.55), with a consistent but non-significant point estimate at 12 months (aHR of 1.90), and with significant 6-month MACE estimate (aHR of 3.07). Erythrocytosis was not associated with mortality, MACE or thrombosis at any timepoint, although the small number of patients and thrombotic events limits interpretation. Dapagliflozin and empagliflozin were associated with indistinguishable hemoglobin trajectories. **Conclusions:** On SGLT2i therapy, anemia carries the dominant prognostic signal and identifies a high-risk subgroup; erythrocytosis is uncommon, transient and showed no detectable association with adverse outcomes, though thrombotic events were too few for firm safety conclusions. Routine monitoring of hemoglobin for anemia is of higher clinical importance compared to erythrocytosis; these single-center findings require external validation.

## 1. Introduction

Sodium-glucose cotransporter 2 inhibitors (SGLT2is) are guideline-recommended medications for the treatment of heart failure (HF) and have been shown to reduce cardiovascular (CV) death and HF hospitalization across the ejection fraction spectrum [1,2,3,4]. A reproducible pharmacologic signature of empagliflozin and dapagliflozin is a rise in hemoglobin and hematocrit on therapy, established in mechanistic and outcome studies [5,6,7,8,9,10]. Three mechanisms have been proposed: an early plasma volume contraction consistent with the natriuretic and osmotic action of SGLT2i [7], a true erythropoietic stimulus through suppression of hepcidin and modulation of iron-regulatory proteins [8], and relief of renal tubular hypoxia with engagement of hypoxia-inducible factor (HIF) signaling [9,10].

Increase in hemoglobin, hematocrit or erythrocyte count may surpass predefined thresholds for chronic myeloproliferative neoplasm (MPN), defined for polycythemia vera (PV) by the WHO and ICC 2022 criteria as >165 g/L in men and >160 g/L in women [11,12]. The clinical threshold for hematological workup in the context of SGLT2i-associated erythrocytosis is uncertain. Equally uncertain is whether SGLT2i can be safely used in patients with established MPN. On the opposite side of the spectrum, some patients develop anemia while already receiving an agent expected to raise hemoglobin, introducing additional clinical dilemmas. While erythrocytosis during SGLT2i treatment has attracted considerable interest [13,14], anemia has received less attention. Several real-world cohorts have characterized hemoglobin dynamics on SGLT2i in type 2 diabetes, chronic kidney disease (CKD) and HF populations and informed our current understanding. In type 2 diabetes, dapagliflozin corrected anemia in 52% of anemic participants (vs. 26% on placebo) in pooled phase 3 data [15], and canagliflozin reduced incident anemia and the use of anemia-related interventions in the CREDENCE trial of diabetic CKD [16]. A large propensity score-matched Israeli cohort confirmed a 5 to 6% absolute excess of new-onset erythrocytosis on SGLT2i compared with DPP-4 inhibitors or GLP-1 receptor agonists, with male sex, smoking and empagliflozin (vs. dapagliflozin) as independent predictors, and no association with arterial or venous thrombotic events [17]. Smaller hematology-referral cohorts described rates of incident erythrocytosis between 2% and 22% on SGLT2i, with thrombotic events in 2.4 to 10% of patients, although baseline thrombotic risk in those populations was not always defined [18,19,20,21]. A recent post hoc analysis of the CANVAS and CREDENCE trials found that canagliflozin raised hematocrit irrespective of baseline level, and that thrombotic risk was concentrated in males with baseline erythrocytosis, an effect not observed in women [22]. A systematic review of drug-induced erythrocytosis confirmed the SGLT2i-class effect and noted that thromboembolic risk in non-randomized SGLT2i series is heterogeneous and confounded by underlying cardiovascular morbidity [23]. Dedicated HF cohorts addressing both anemia and erythrocytosis on SGLT2i remain scarce.

In the present study we analyzed a large prospective institutional registry of HF patients and described baseline characteristics across three clinically interpretable hemoglobin categories (normal, anemia and erythrocytosis), modeled patient-level transitions between categories at three timepoints (baseline, 6 and 12 months), identified univariable and multivariable predictors of erythrocytosis and of anemia at each timepoint using backwards stepwise regression, and performed landmark survival analyses for time to death, MACE and thrombosis accounting for the competing risk of death.

## 2. Materials and Methods

### 2.1. Study Design and Cohort

The Cardiology Registry of University Hospital Dubrava is a prospective, single-center observational registry of patients with chronic or acute HF managed at the Department of Cardiology of University Hospital Dubrava (Zagreb, Croatia). Patients who consecutively initiated dapagliflozin 10 mg or empagliflozin 10 mg between March 2022 and October 2025 were eligible for the present analysis. The study was approved by the institutional ethics committee (approval 2022/1403-01, 14 March 2022) and conducted in accordance with the Declaration of Helsinki [24]. The registry was retrospectively registered on ClinicalTrials.gov (NCT06090591) on 13 October 2023, after enrollment had begun, with no subsequent changes to the pre-specified protocol. All participants gave informed consent.

### 2.2. Variables and Follow-Up

Demographics, comorbidities, concomitant medications, vital signs, laboratory values, echocardiographic ejection fraction (LVEF) and NT-proBNP were recorded at baseline (the index visit at which SGLT2i was initiated) and at scheduled follow-up visits at 6 and 12 months. Adherence to SGLT2i was scored using the 8-item Morisky Medication Adherence Scale (MMAS-8) [25] and categorized as high (score 8), moderate (6–7), low (<6), or not taking. Discontinuation reasons were captured. Follow-up hemoglobin values were not available for all patients, predominantly due to death during study and due to back-referral of patients to their primary or regional institutions.

### 2.3. Hemoglobin Status Categorization

At each timepoint patients were categorized by hemoglobin concentration (g/L) using sex-specific thresholds. Anemia was defined as <120 g/L in women and <130 g/L in men, following the conventional World Health Organization (WHO) sex-specific hemoglobin thresholds for the diagnosis of anemia [26]. Erythrocytosis was defined as >160 g/L in women and >165 g/L in men, reflecting the major hemoglobin criterion for polycythemia vera in the WHO 5th edition and in the International Consensus Classification of myeloid neoplasms [11,12]. Categorization was based on hemoglobin due to available practical definitions established for other medical contexts, i.e., categories are pragmatic clinical definitions rather than strict hematologic diagnoses; a similar approach was used in prior studies [27,28]. In a small proportion of patients with discordant hemoglobin/hematocrit/RBC values category allocation was based on hemoglobin concentration. Patients with pre-existing or newly diagnosed MPNs were excluded by review of clinical records.

### 2.4. Outcomes

Outcomes were obtained from electronic medical records and, where necessary, direct contact with the patient or family physician. All-cause death was the primary mortality endpoint. Major adverse cardiovascular events (MACEs) were defined as the composite of HF decompensation requiring hospitalization, acute coronary syndrome, cerebrovascular events (stroke or transient ischemic attack), or thromboembolic or peripheral vascular events. Thrombotic events comprised acute coronary syndromes, ischemic stroke and venous or peripheral arterial thrombotic events. The date of the first event was recorded.

### 2.5. Statistical Analysis

Continuous variables are presented as median (interquartile range) and compared across hemoglobin categories using the Kruskal–Wallis test. Categorical variables are presented as count (%) and compared across categories with chi-square or Fisher’s exact test. Hemoglobin transitions across timepoints were displayed using three complementary visualizations: a composite three-tier alluvial diagram including all enrolled patients with a missing category for patients without a measurement at a given visit, row-percentage transition heatmaps for the three pairwise transitions, and individual-level hemoglobin trajectories with overlaid median curves and a stacked bar count panel.

Predictors of erythrocytosis vs. normal and of anemia vs. normal hemoglobin were assessed at each of the three timepoints. For each outcome and each timepoint, we first performed univariable logistic regression for a pre-specified panel of candidate variables. The candidate set comprised demographic, comorbidity, laboratory, hemodynamic and treatment-related variables: age, sex, body mass index, current smoking, diabetes mellitus, hypertension, dyslipidemia, atrial fibrillation, coronary artery disease, prior myocardial infarction, peripheral arterial disease, chronic kidney disease, COPD or asthma, prior stroke, HF phenotype (HFrEF vs. HFmrEF vs. HFpEF), left ventricular ejection fraction, NT-proBNP, estimated glomerular filtration rate, serum sodium, serum potassium, serum albumin, C-reactive protein, red cell distribution width, MMAS-8 adherence category, and SGLT2i agent (dapagliflozin vs. empagliflozin). Multivariable logistic regression was performed separately at each timepoint, including all variables univariably associated with the outcome at that timepoint at *p* < 0.10; for the 6- and 12-month models the baseline hemoglobin value (continuous) was additionally forced into the multivariable models. Predictors retained at *p* < 0.05 in the multivariable model are reported as independent. A backward-selection strategy with forced inclusion of baseline hemoglobin was chosen owing to the exploratory, hypothesis-generating nature of the current study, and was deemed most suitable for identifying independent predictors among a large number of candidate variables. As a sensitivity analysis, the 6-month erythrocytosis and anemia models were internally validated by bootstrapping (1000 replicates; optimism-corrected c-statistic and calibration slope) and re-estimated with penalized (LASSO and Firth) logistic regression.

For survival analyses, a landmark approach was used to avoid immortal-time bias: at each of three landmark times (baseline, 6 months, 12 months) the cohort was restricted to patients at risk at the landmark, and hemoglobin category at the landmark was the exposure of interest. The 6- and 12-month landmarks were chosen because they coincide with the pre-scheduled registry follow-up visits, align with the guideline-recommended intervals for clinical and laboratory reassessment of heart failure, and span the period over which the hematologic effect of SGLT2i is established and plateaus. For all-cause death Kaplan–Meier curves, log-rank test and Cox proportional hazards models (univariable and multivariable, adjusted for age, sex, LVEF, eGFR and NT-proBNP) were used. For MACE and thrombosis cumulative incidence functions accounting for the competing risk of death and reported cause-specific Cox hazard ratios with cause-specific *p*-values were estimated [29]. As a sensitivity analysis, hemoglobin status was modeled as a time-varying covariate in a single Cox model with three exposure intervals per patient. Given prior evidence of a male-specific thrombotic signal of erythrocytosis on SGLT2i [22], a pre-specified post hoc analysis of the sex-by-erythrocytosis interaction for thrombotic events was also performed.

The relationship between change in hemoglobin and change in LVEF or NT-proBNP at the same timepoint was assessed using Spearman correlation, by group comparisons across categorical Hgb-trajectory direction (upward vs. stable vs. downward) with Kruskal–Wallis and Mann–Whitney tests, and by the binary dichotomy delta-Hgb > 0 vs. delta-Hgb ≤ 0. NT-proBNP changes were log-transformed before correlation. Within-patient changes in proportion of a given hemoglobin category between consecutive timepoints were tested with the McNemar test for paired binary outcomes, restricted to patients with measurements at both timepoints. Subgroup analyses by SGLT2i agent (dapagliflozin vs. empagliflozin) were performed for category distributions at each timepoint, de novo erythrocytosis and resolution of baseline erythrocytosis. Two-sided *p* < 0.05 was considered statistically significant. Analyses were performed in Python version 3.11 (Python Software Foundation, Beaverton, OR, USA) using the statsmodels library version 0.14, the lifelines library version 0.27, and the SciPy library version 1.11 (all open-source scientific Python libraries) and MedCalc Statistical Software version 23.5.2 (MedCalc Software Ltd., Ostend, Belgium).

## 3. Results

### 3.1. Cohort Characteristics

The analytic cohort comprised 1244 patients with a baseline hemoglobin measurement. The median age was 69 years (IQR 61–76); 33.8% were female; and 50.4% received dapagliflozin and 49.6% received empagliflozin. The HF phenotype distribution was: HFrEF 58.3%, HFmrEF 15.4%, and HFpEF 26.4%. Hypertension (85%), coronary artery disease (54.2%), and diabetes (41.4%) were the most prevalent comorbidities. The median LVEF was 40% (IQR 30–50) and the median NT-proBNP level was 2419 pg/mL (988–6173).

SGLT2is were discontinued during the study period in 160 patients (12.9%), and the reasons in descending order of frequency were: the patient felt it was no longer needed (22.5%), intolerance or adverse effects other than urinary tract infections (19.4%), urinary tract infections (17.5%), out-of-pocket cost (15.6%), worsening renal function (10.0%), physician-directed withdrawal by an endocrinologist, cardiologist or GP (8.1%), and other reasons including post-transplantation, hospitalization, electrolyte imbalance or unspecified (5.6%), whereas erythrocytosis accounted for only two discontinuations.

At baseline, 23.6% (n = 294) had anemia, 71.1% (n = 884) were normal, and 5.3% (n = 66) had erythrocytosis. The three groups differed substantially across nearly every measured domain (Table 1). Anemic patients were older, with worse renal function, higher NT-proBNP, higher CRP, lower albumin and lower lipid fractions. Erythrocytic patients were younger, predominantly male (84.8%), and more often current smokers, with lower LVEF and higher heart rate. Their NT-proBNP and HF phenotype distribution were similar to normal-hemoglobin patients.

### 3.2. Hemoglobin Trajectories and Inter-Category Transitions

Median hemoglobin rose from 138 g/L at baseline to 140 g/L at 6 months and 142 g/L at 12 months (Wilcoxon signed-rank *p* < 0.001 for baseline vs. 6 months and baseline vs. 12 months among paired measurements). Across paired timepoints the proportion of patients in the erythrocytosis category rose significantly from baseline to 6 months and then returned toward baseline by 12 months (5.3% at baseline, 8.0% at 6 months, 5.9% at 12 months, with each expressed as a percentage of patients with an available measurement at that visit; McNemar paired-proportion test *p* < 0.001 for baseline vs. 6 months, and *p* = 0.46 for baseline vs. 12 months), while the proportion in the anemia category decreased significantly (23.6%, 20.6%, 17.6%; McNemar *p* = 0.016 for baseline vs. 6 months and *p* = 0.002 for baseline vs. 12 months). These prevalence figures, however, obscure considerable patient-level movement between categories. Per-patient change in hemoglobin from baseline (delta-Hgb) followed the expected pattern across the three follow-up status categories. Overall, the median delta-Hgb among patients with paired measurements was +3.0 g/L (IQR −4.0 to +10.5, n = 927) at 6 months and +4.0 g/L (IQR −4.0 to +12.0, n = 784) at 12 months. At the 6-month visit, patients ending in the erythrocytosis category had a median delta-Hgb of +14.5 g/L (IQR +5.2 to +21.0, n = 74), patients ending normal +3.0 g/L (IQR −3.0 to +10.0, n = 662), and patients ending anemic −2.0 g/L (IQR −13.0 to +6.0, n = 191), where Kruskal–Wallis *p* < 0.001. At the 12-month visit, the corresponding medians were +18.0 g/L (IQR +9.2 to +26.8, n = 46) in erythrocytosis, +4.0 g/L (IQR −2.0 to +12.0, n = 600) in normal, and −4.0 g/L (IQR −16.0 to +6.0, n = 138) in anemia, where Kruskal–Wallis *p* < 0.001.

**Figure 1:**
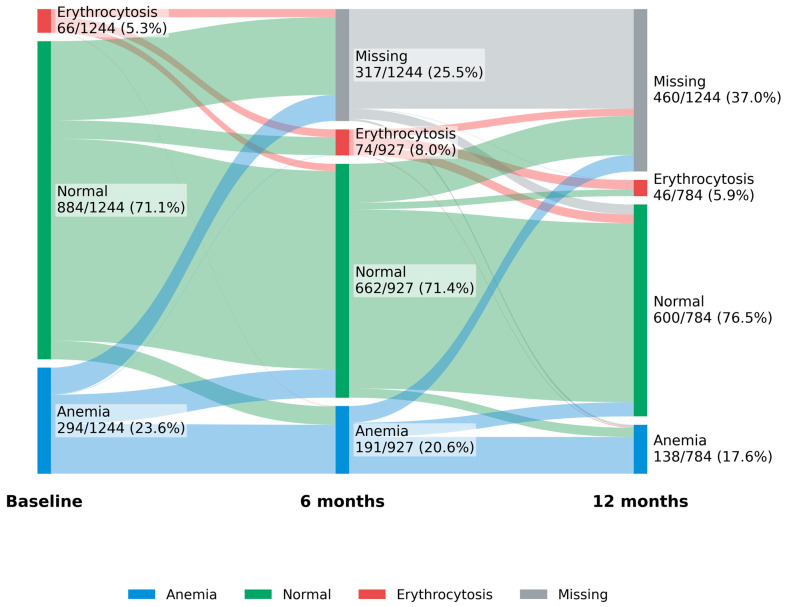
Hemoglobin status transitions across baseline, 6 months and 12 months. The missing category is expressed as a percentage of the full 1244-patient cohort, whereas the anemia, normal and erythrocytosis categories are expressed as percentages of the patients with an available measurement at that visit (n = 1244 at baseline, 927 at 6 months and 784 at 12 months).

**Figure 2:**
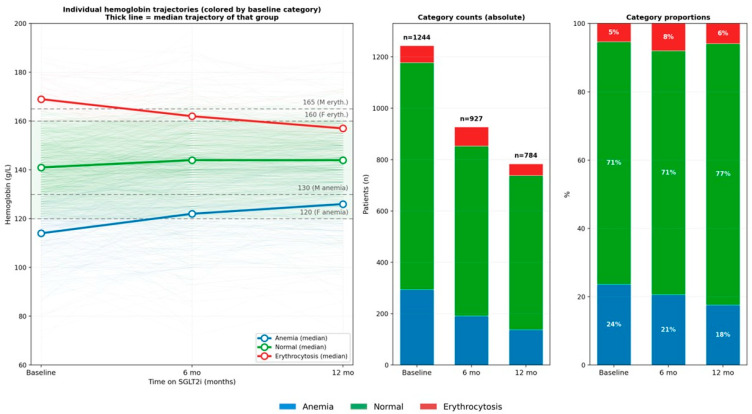
Individual hemoglobin trajectories by baseline category. Individual hemoglobin trajectories are colored by baseline category, with overlaid median trajectories per group and a stacked-bar panel showing counts and proportions per timepoint. Dashed reference lines mark the sex-specific thresholds (120/130 g/L for anemia in women/men, 160/165 g/L for erythrocytosis in women/men).

Inter-category transitions are displayed in the alluvial diagram in Figure 1 and summarized in the row-percentage heatmaps of Supplementary Figure S1. The great majority of baseline-normal patients remained in the normal stratum (84% at 6 months, 85% at 12 months from baseline, 92% over the 6- to 12-month interval, n = 749). At 6 months, among 664 baseline-normal patients with a measurement, 52 (7.8%) had become anemic and 51 (7.7%) had become erythrocytic, with the upward and downward transitions occurring at essentially identical rates. By 12 months the balance of transition from normal shifted further toward anemia: of 568 baseline-normal patients with a measurement, 52 (9.2%) had become anemic versus 34 (6.0%) erythrocytic. Among baseline-erythrocytic patients, 47% returned to normal by 6 months and 72% by 12 months, with the remaining patients staying erythrocytic and a negligible number transitioning to anemia. Among baseline-anemic patients, 37% resolved to normal by 6 months and 51% by 12 months, with the remainder staying anemic. Among the 749 patients with hemoglobin measurements at all three timepoints, nine (1.2%) were continuously in the erythrocytosis category at baseline, 6 and 12 months, and 26 (3.5%) were in the erythrocytosis category at both the 6- and 12-month visits irrespective of baseline status (16 of these 26 had transitioned upward from baseline normal, nine had remained erythrocytic since baseline, and one had transitioned from baseline anemia). The spaghetti plot of individual trajectories (Figure 2) suggests that within each baseline category there is a wide range of individual trajectories and that the median trajectories of the three categories converge towards the normal band by 12 months. The downward flow from the normal tier is therefore quantitatively as important as, and by 12 months exceeds, the upward flow to erythrocytosis that is often the focus of clinical concern. However, all three categories tended toward normalization of hemoglobin values over time.

We additionally assessed whether upward hemoglobin trajectories were accompanied by concurrent improvement in LVEF or in NT-proBNP. Across the entire analytic cohort, change in hemoglobin showed no association with change in LVEF at either 6 months (Spearman ρ = +0.03, *p* = 0.340) or 12 months (ρ = +0.04, *p* = 0.220). In contrast, increasing hemoglobin was significantly associated with a greater fall in NT-proBNP (ΔlogNT-proBNP) at both 6 months (ρ = −0.13, *p* < 0.001) and 12 months (ρ = −0.15, *p* < 0.001). The same pattern was apparent when patients were grouped by categorical Hgb-trajectory direction: those undergoing an upward transition had a larger median fall in logNT-proBNP than those with stable or downward transitions (−1.1 vs. −0.7 at 6 months; Mann–Whitney *p* = 0.033), with a consistent trend at 12 months, but no detectable difference in ΔLVEF. A dichotomy of ΔHgb > 0 vs. ≤0 (positive in 60.0% of patients at 6 months and 61.2% at 12 months) was in line with aforementioned analyses: patients with an Hgb increase had a greater median NT-proBNP reduction (ΔlogNT-proBNP −0.85 vs. −0.62 at 6 months, *p* = 0.001; −1.09 vs. −0.78 at 12 months, *p* = 0.010), with only a non-significant trend toward higher ΔLVEF. Together these data suggest that the SGLT2i-associated hemoglobin rise tracks more closely with hemodynamic and natriuretic-peptide decongestion than with structural recovery of systolic function. Because hemoglobin and NT-proBNP were measured concurrently, the causal direction of this association cannot be established from our dataset.

### 3.3. Predictors of Erythrocytosis vs. Normal Hemoglobin at Three Timepoints

Univariable logistic regressions of erythrocytosis vs. normal hemoglobin were performed at baseline, 6 months and 12 months. At baseline variables univariably associated with erythrocytosis at *p* < 0.05 were younger age, current smoking, lower LVEF, higher heart rate, male sex, and absence of hypertension. At 6 months univariable predictors at *p* < 0.05 included higher baseline hemoglobin, current smoking, COPD or asthma, and lower serum albumin, and male sex. At 12 months univariable predictors at *p* < 0.05 included higher baseline hemoglobin and male sex. The full list of candidate variables and their univariable estimates at each timepoint is provided in Supplementary Table S1.

In the multivariable model at baseline, current smoking (adjusted OR (aOR) 2.43, 95% CI 1.18 to 5.02, *p* = 0.016) and higher heart rate (aOR 1.19 per +10 bpm, 1.05 to 1.35, *p* = 0.007) were jointly retained as independent predictors of baseline erythrocytosis.

In the multivariable model at 6 months, higher baseline hemoglobin (aOR 3.11 per +10 g/L, 95% CI 2.37 to 4.41, *p* < 0.001), non-female sex (aOR 0.31, 0.14 to 0.69, *p* = 0.004, protective), current smoking (aOR 1.92, 1.10 to 3.36, *p* = 0.022), COPD or asthma (aOR 2.06, 1.05 to 4.05, *p* = 0.036), lower serum albumin (aOR 0.56 per +5 g/L, 0.41 to 0.77, *p* < 0.001) and high adherence (high vs. moderate; aOR 2.86, 1.08 to 7.69, *p* = 0.035) were jointly retained as independent predictors of 6-month erythrocytosis.

In the multivariable model at 12 months, higher baseline hemoglobin (aOR 1.97 per +10 g/L, 95% CI 1.48 to 2.59, *p* < 0.001) and non-female sex (aOR 0.16, 0.03 to 0.85, *p* = 0.031, protective) were jointly retained as independent predictors of 12-month erythrocytosis.

### 3.4. Predictors of Anemia vs. Normal Hemoglobin at Three Timepoints

Univariable logistic regressions of anemia vs. normal hemoglobin were performed at baseline, 6 months and 12 months. At baseline, variables univariably associated with anemia at *p* < 0.05 included older age, diabetes, hypertension, COPD or asthma, peripheral arterial disease, chronic kidney disease, higher NT-proBNP, lower eGFR, higher CRP, higher RDW, lower albumin, lower BMI, absence of current smoking, and dapagliflozin vs. empagliflozin. At 6 months, univariable predictors of anemia at *p* < 0.05 included lower baseline hemoglobin, older age, diabetes, peripheral arterial disease, chronic kidney disease, COPD or asthma, atrial fibrillation, prior stroke, higher NT-proBNP, lower eGFR, higher CRP, higher RDW, higher serum potassium, lower serum sodium, lower albumin, lower BMI, and low adherence. At 12 months, univariable predictors of anemia at *p* < 0.05 included lower baseline hemoglobin, older age, diabetes, peripheral arterial disease, chronic kidney disease, HFpEF/HFmrEF phenotype, higher LVEF, higher NT-proBNP, lower eGFR, higher RDW, higher serum potassium, lower serum sodium, lower albumin, and low adherence. The full list of candidate variables and their univariable estimates at each timepoint is provided in Supplementary Table S2.

In the multivariable model at baseline, diabetes (aOR 1.57, 95% CI 1.02 to 2.42, *p* = 0.039), coronary artery disease (aOR 1.70, 1.10 to 2.62, *p* = 0.016), higher LVEF (aOR 1.66 per +10%, 1.39 to 1.98, *p* < 0.001), higher NT-proBNP (aOR 1.06 per +1000 pg/mL, 1.02 to 1.09, *p* < 0.001), lower eGFR (aOR 0.82 per +10 mL/min/1.73 m^2^, 0.74 to 0.91, *p* < 0.001), lower albumin (aOR 0.61 per +5 g/L, 0.47 to 0.77, *p* < 0.001) and higher RDW (aOR 1.81 per +1%, 1.57 to 2.09, *p* < 0.001) were jointly retained as independent predictors of baseline anemia.

In the multivariable model at 6 months, lower baseline hemoglobin (aOR 0.43 per +10 g/L, 95% CI 0.35 to 0.54, *p* < 0.001), peripheral arterial disease (aOR 2.77, 1.39 to 5.54, *p* = 0.004), lower albumin (aOR 0.71 per +5 g/L, 0.55 to 0.92, *p* = 0.010), low adherence (low vs. high; aOR 2.13, 1.02 to 4.45, *p* = 0.044) and the ‘not taking’ category (vs. high; aOR 6.58, 2.49 to 17.38, *p* < 0.001) were jointly retained as independent predictors of 6-month anemia.

In the multivariable model at 12 months, lower baseline hemoglobin (aOR 0.54 per +10 g/L, 95% CI 0.43 to 0.66, *p* < 0.001), older age (aOR 1.97 per +10 years, 1.23 to 3.15, *p* = 0.005) and low or absent adherence (low or ‘not taking’ vs. high; aOR 3.98, 1.28 to 12.37, *p* = 0.017) were jointly retained as independent predictors of 12-month anemia.

### 3.5. Landmark Survival Analyses

**Figure 3:**
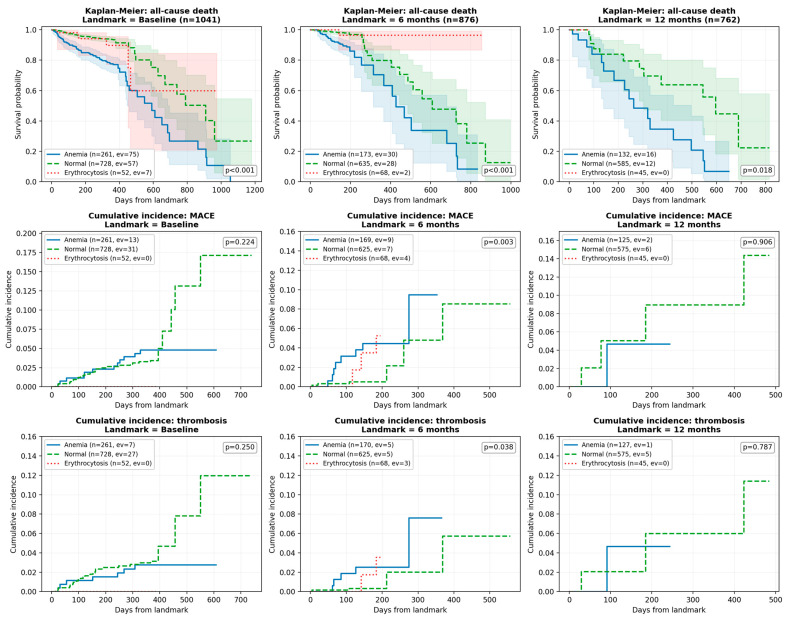
Landmark analyses for clinical outcomes stratified by hemoglobin status. Rows: outcomes (all-cause death, MACE, thrombosis). Columns: landmark times (baseline, 6 months, 12 months). For death, Kaplan–Meier curves are shown; for MACE and thrombosis, cumulative incidence functions accounting for death as a competing risk are shown with cause-specific log-rank *p*-values. Shaded areas around the Kaplan–Meier curves represent 95% confidence intervals.

Over a median follow-up of 365 days from SGLT2i initiation, 140 of the 1244 patients in the analytic cohort (11.3%) died, 46 (3.7%) experienced a MACE event, and 35 (2.8%) experienced a thrombotic event. Kaplan–Meier estimates of all-cause survival and cumulative incidence functions for MACE and thrombosis at each landmark are shown in Figure 3.

Anemia at the landmark was strongly and consistently associated with all-cause death. Baseline anemia conferred an adjusted HR of 2.23 (95% CI 1.54–3.22; *p* < 0.001) relative to normal; 6-month anemia conferred an aHR of 2.45 (1.45–4.16; *p* = 0.001); and 12-month anemia conferred an aHR of 1.90 (0.89–4.07; *p* = 0.097). Erythrocytosis at the landmark was not associated with death at any timepoint (baseline aHR of 1.27, 0.59–2.74; 6-month aHR 0.74, 0.22–2.49; 12-month aHR of 0.76, 0.05–11.50). Detailed Cox estimates are provided in Supplementary Table S3, and a sensitivity analysis modeling hemoglobin status as a time-varying covariate (yielding a HR of 2.55, 1.80–3.62 for anemia and a HR of 1.20, 0.52–2.72 for erythrocytosis) is given in Supplementary Table S4.

Anemia at the 6-month landmark was also independently associated with MACE (aHR of 3.07, 1.27 to 7.38, *p* = 0.012), and there was a non-significant trend at 6 months for thrombosis (univariable HR of 3.27, 0.97 to 11.07, *p* = 0.057). The MACE association at baseline was non-significant (aHR of 1.08, 0.58 to 2.02). Erythrocytosis was not associated with MACE or thrombosis at any landmark. In a pre-specified post hoc analysis of the sex-by-erythrocytosis interaction for thrombotic events, the absolute number of thrombotic events in the erythrocytic subgroup was small and no formal interaction test could be reliably performed, and restricting to males, the cumulative incidence of thrombosis was not significantly different from normal men.

### 3.6. Empagliflozin vs. Dapagliflozin

The two SGLT2i agents differed slightly in baseline Hgb-category distribution (more anemia in the dapagliflozin arm, 26.6% vs. 20.8%, chi-square *p* = 0.029) but became indistinguishable at 6 months (*p* = 0.368) and 12 months (*p* = 0.834). Median hemoglobin rose comparably with both drugs at every timepoint. Among baseline-normal patients, de novo erythrocytosis at 6 months occurred in 7.4% on dapagliflozin and 8.0% on empagliflozin (Fisher *p* = 0.773); the corresponding 12-month rates were 6.5% and 5.4% (*p* = 0.602), Supplementary Figure S2. Resolution of baseline erythrocytosis was similar between the drugs (48% vs. 50% at 6 months, 78% vs. 69% at 12 months). Similarly for anemia development, among baseline-normal patients de novo anemia at 6 months occurred in 8.9% on dapagliflozin and 6.8% on empagliflozin (Fisher *p* = 0.386) and at 12 months in 9.9% on dapagliflozin and 8.3% on empagliflozin (*p* = 0.562). Resolution of baseline anemia was likewise comparable between the drugs (38% vs. 37% at 6 months, 57% vs. 46% at 12 months). In a time-varying Cox model for all-cause death with a drug-by-hemoglobin-status interaction term, no interaction reached statistical significance, indicating that the prognostic implications of hemoglobin status are similar across the two agents.

### 3.7. Additional Considerations

Because follow-up hemoglobin was unavailable in a substantial proportion of patients (25.5% at 6 months and 37.0% at 12 months), we compared the baseline characteristics of patients with and without an available 12-month measurement across the recorded clinical variables (Supplementary Table S5). Patients without 12-month hemoglobin data were older (median 71 vs. 69 years), more tachycardic (88 vs. 82 bpm), and had higher NT-proBNP (3040 vs. 2064 pg/mL), lower serum sodium and lower serum albumin (all *p* < 0.05), a profile of more advanced, more congested heart failure. On the other hand, baseline hemoglobin (138 vs. 139 g/L, *p* = 0.56), the categorical hemoglobin distribution, eGFR, LVEF, CRP and RDW did not differ between the groups. Missingness was therefore not completely random with respect to heart-failure severity, yet was non-differential with respect to the exposure of interest. Because the patients lost to follow-up were on average sicker, and because death (a competing event) contributed to the attrition, the 12-month landmark and late-transition estimates are expected to be biased toward the null, consistent with the attenuated, non-significant 12-month anemia hazard ratio. The absence of a baseline-hemoglobin difference argues against differential misclassification of hemoglobin status.

To examine whether the prognostic weight of anemia depends on its persistence, we classified patients with a baseline and at least one follow-up measurement as having persistent, resolved or new-onset anemia, with stable non-anemic patients shown for reference (Supplementary Table S6). Persistent anemia (n = 115) identified the oldest (median 75 years) and most comorbid subgroup, with the highest RDW (15.4%), the lowest eGFR (51 mL/min/1.73 m^2^), and the highest burden of chronic kidney disease, peripheral arterial disease and diabetes. The trajectories were separated principally by age, heart rate, eGFR, serum sodium, RDW and baseline hemoglobin, but not by NT-proBNP, serum albumin, CRP or most comorbidities, suggesting that they reflect differences in chronicity and renal-erythropoietic reserve rather than in acute congestion. In a 6-month landmark Cox analysis, all three anemia trajectories carried a significantly higher adjusted hazard of death than stable non-anemic patients (persistent aHR of 2.93, 95% CI 1.51–5.67; new-onset 4.40, 1.74–11.17; resolved 2.35, 0.98–5.66; adjusted for age, sex, LVEF, eGFR and NT-proBNP), with no significant difference among the three anemia groups. Because these groups are defined after baseline, the comparison is descriptive and cannot fully separate the prognostic effect of anemia from the underlying disease severity that drives both; the small number of deaths within each anemia trajectory also limits power to distinguish them.

As a sensitivity analysis addressing the stability of the backwards-selected models, the two 6-month models were internally validated by bootstrapping and re-estimated with penalized regression. The anemia model was robust (optimism-corrected c-statistic of 0.86, calibration slope pf 0.95), with low baseline hemoglobin, peripheral arterial disease and non-adherence selected in 72–100% of bootstrap resamples and concordant across backward, LASSO and Firth estimations. In the low-event erythrocytosis model only baseline hemoglobin was a robust predictor (optimism-corrected c-statistic 0.83), whereas the secondary predictors (current smoking, COPD, sex and adherence) were selected inconsistently and attenuated under penalization and should be regarded as exploratory.

## 4. Discussion

There are several key points we would like to emphasize, based on the currently presented analyses of our prospective single-center registry of 1244 HF patients receiving SGLT2i therapy. First, anemia is the hemoglobin category that carries the strongest and most consistent prognostic signal. Anemia identified a subgroup with approximately 2- to 2.5-fold higher all-cause mortality at the baseline and 6-month landmarks (statistically non-significant at 12 months), and at 6 months it also identified patients with markedly higher subsequent MACE. Second, erythrocytosis was comparatively uncommon (around 5–8% at any visit), with substantial de novo development and substantial resolution. In this cohort it was not associated with adverse outcomes at any timepoint. Third, in multivariable backwards models, the patient’s own baseline hemoglobin value predicted subsequent erythrocytosis and subsequent anemia most consistently and with some of the most pronounced associations among investigated variables. In practical terms, hemoglobin concentration at SGLT2i initiation is the single most informative parameter for where it will be at 6 and 12 months, suggesting that intrinsic individual erythropoietic biology dominates over modifiable treatment factors in shaping its subsequent trajectory. Fourth, baseline-normal patients transitioned downward to anemia at rates equal to or exceeding the rate of upward transition to erythrocytosis, with downward transition characterized by peripheral arterial disease, advanced metabolic comorbidity and pharmacologic non-adherence. Fifth, dapagliflozin and empagliflozin showed hematologically indistinguishable trajectories in this cohort, both for erythrocytosis and for anemia development and resolution.

Observed absolute hemoglobin findings in the current study are similar in direction and magnitude to those discussed in the foundational randomized SGLT2i trials in HF [1,2,3,4]. Mechanistically, the SGLT2i-associated hemoglobin rise is widely interpreted as reflecting several convergent processes [5,6,7,8,9,10,30]. The mediation analysis of EMPA-REG OUTCOME indicates that changes in hematocrit and hemoglobin themselves mediate approximately half of the empagliflozin-attributable reduction in CV death (51.8% and 48.9%, respectively) [5]. The plasma volume contraction secondary to osmotic and natriuretic diuresis is proposed as an early mechanism associated with erythrocytosis [7]. A more sustained mechanism is restoration of iron utilization with a modest rise in endogenous erythropoietin. In a randomized study of dapagliflozin in obese type 2 diabetes, hepcidin and ferritin were significantly reduced and erythropoietin transiently increased on therapy [8]. Maruyama et al. showed an approximately 38% rise in serum erythropoietin within 2 to 4 weeks of canagliflozin in diabetic CKD patients with anemia [10], and Mazer et al. similarly documented an early erythropoietin and reticulocyte rise with empagliflozin in coronary artery disease patients with type 2 diabetes [6]. Packer has argued that SGLT2is alleviate renal tubular hypoxia and shift the HIF-1α to HIF-2α balance toward erythropoietin production [9]. Real-world cohorts have characterized these effects across HF, diabetes and CKD populations: a modest mean hemoglobin rise (in the order of 4 g/L) on SGLT2is versus comparators, marginally larger with empagliflozin than dapagliflozin [17,20], together with reduced incident anemia and correction of anemia in roughly half of anemic patients in diabetic CKD and pooled diabetes datasets [15,16]. Hematology-referral series described peak incident erythrocytosis between 2% and 22% on SGLT2i and a heterogeneous thrombotic event rate of 2.4 to 10% [18,19,21], and a recent comprehensive review of drug-induced erythrocytosis concluded that drug discontinuation purely for asymptomatic erythrocytosis is not warranted [23,30]. Together, these mechanisms and cohorts predict that patients with intact erythropoietic capacity (younger, less inflamed, with adequate iron handling and without advanced vasculopathy) are able to mount a hemoglobin rise on SGLT2i and they constitute the bulk of those crossing the erythrocytosis threshold. Patients lacking that capacity would fail to do so, and a subset would regress to anemia.

The first key point concerns anemia and prognosis. Anemia and iron deficiency in HF have long been associated with worse clinical status and outcomes [31], and our data extend this association to the SGLT2i context. The robust 2- to 2.5-fold mortality signal at the baseline and 6-month landmarks, the consistent effect size in the time-varying Cox model, and the strong 6-month MACE association argue that patients who remain anemic or become anemic on SGLT2i deserve careful consideration. The CREDENCE post hoc analysis [16] and the dapagliflozin pooled analysis of Stefánsson [15] both showed that SGLT2is correct anemia in a substantial proportion of anemic diabetic patients (52% on dapagliflozin vs. 26% on placebo in the pooled phase 3 dapagliflozin data), comparable to the 37% baseline-anemia-to-normal transition we observed at 6 months. The clinically important aspect is the residual group, those who remain anemic or who become anemic despite a hemoglobin-raising drug, and our data identify this group as prognostically relevant. Whether residual anemia reflects unmasking of intrinsic erythropoietic failure, absolute or functional iron deficiency that overrides the SGLT2i effect, advanced renal dysfunction, chronic inflammation/cardiorenal anemia, haemodilution, or simply the severity of the underlying HF cannot be concluded from the present data and further studies in this regard are required. Anemia etiologies were not, however, systematically protocolized in our registry and were captured from clinical records, which we acknowledge as a limitation. The 6-month MACE association being stronger than the baseline association suggests that anemia developing on therapy carries stronger event implications than anemia present at the moment of SGLT2i initiation. Consistent with this, persistent anemia in our cohort identified the oldest and most comorbid subgroup, yet in a 6-month landmark analysis each trajectory (persistent, new-onset and resolved) carried a significantly higher adjusted mortality than stable non-anemic patients, with no significant difference among the three and with new-onset anemia at least as adverse as persistent anemia.

The second point is the apparent prognostic neutrality, in this cohort, of SGLT2i-associated erythrocytosis. The pivotal evidence linking elevated hematocrit to thrombosis derives from polycythemia vera. In the CYTO-PV randomized trial, patients with PV randomized to a hematocrit target below 45% experienced significantly fewer composite events (cardiovascular death plus major thrombosis) than those with a target of 45 to 50% [32]. That signal motivated parallel concern for SGLT2i-associated hemoglobin rise, reported by Tukker et al. as severe dapagliflozin-associated polycythemia [14], extended by the Rotterdam group to a multicenter real-world series [13]. Three large analyses do not support a clinically meaningful thrombotic signal of SGLT2i-associated erythrocytosis at the population level. The propensity score-matched Israeli cohort of Lewis et al. reported no increased risk of myocardial infarction (HR of 0.92), venous thromboembolism (HR of 1.56) or stroke (HR of 1.26) in patients developing new-onset erythrocytosis on SGLT2i [17]. The systematic review of drug-induced erythrocytosis concluded that a clear causal link to thrombosis has not been established for SGLT2i [23]. The post hoc CANVAS and CREDENCE analysis of Doi et al. is the principal exception, identifying a sex-specific signal where canagliflozin-treated males with baseline erythrocytosis had a higher thrombotic event rate, an effect absent in women [22,30]. Due to the low number of events in the erythrocytic stratum, the present data are best interpreted as showing no detectable adverse signal rather than as positively establishing safety. Combined with the high spontaneous resolution rate (around 50% by 6 months and around 70% by 12 months), the present data also argue against routine drug discontinuation or empirical phlebotomy for an isolated hemoglobin rise on SGLT2i in the absence of other features that would prompt MPN work-up. Of note, real-world clinician behavior in our cohort already reflects this principle, as only two patients (1.2% of all discontinuations and 0.16% of the analytic cohort) were discontinued specifically because of erythrocytosis.

The third point concerns predictors of erythrocytosis and of anemia. The predictor profile we identified for sustained erythrocytosis (male sex, current smoking, higher heart rate, univariably lower LVEF at baseline, and higher baseline hemoglobin at follow-up landmarks) mirrors the male, smoker, empagliflozin-treated profile reported by Lewis et al. [17] and the JAK2-unmutated erythrocytosis literature [30,33]. The mirror-image predictor profile for anemia (older age, diabetes, peripheral arterial disease, low albumin, low eGFR, low adherence, lower baseline hemoglobin) likewise aligns with the iron-deficient, inflamed, cardiorenal phenotype characterized in HF anemia reviews [31].

The fourth point concerns inter-category dynamics. Among baseline-normal patients, the proportion that became anemic on therapy (7.8% at 6 months and 9.2% at 12 months) was as large as or larger than the proportion that developed erythrocytosis (7.7% and 6.0%, respectively). For the majority of patients, however, the average tendency was toward stabilization. Approximately half of baseline-erythrocytic patients and approximately 37% of baseline-anemic patients reverted to normal within 6 months, with the majority of baseline-normal patients (84% at 6 months, 92% over the 6 to 12 month interval) remaining stable. This pattern of net stabilization with bidirectional flow is consistent with the broader observation that SGLT2is tend to recenter the hemoglobin distribution rather than shift the entire cohort in one direction [15,16,17]. Given that anemia rather than erythrocytosis is the prognostically loaded phenotype on SGLT2i in our data, the downward transition is arguably the more important signal for the clinicians and is associated with a modifiable predictor (adherence).

The fifth point concerns differences between empagliflozin and dapagliflozin. In our cohort, hemoglobin trajectories, incident erythrocytosis, incident anemia, resolution rates and prognostic implications were indistinguishable between the two agents. The marginally higher rate of new-onset erythrocytosis with empagliflozin reported in the Israeli cohort [17] and in meta-analyses [34] was not reproduced in our HF population. However, outside of hemoglobin handling, the two drugs may differ in other parameters relevant for HF, diabetes and CKD treatment [35,36].

Translating these observations into hematology clinical practice, several related questions converge. The first is who, among patients on SGLT2i, warrants hematology referral for erythrocytosis. The second is whether established MPN patients with coexisting type 2 diabetes, CKD or HF, for whom SGLT2i would otherwise be indicated, can safely receive these drugs. The third is which tail of the hemoglobin response should drive surveillance, given that our data identify failure to correct, or new development of, anemia as at least as prognostically important as the erythrocytosis that has dominated recent attention. For the first question, the published literature converges on a restrictive approach. Routine referral is likely not warranted for an isolated, single-timepoint hemoglobin above WHO PV thresholds, because approximately half of such patients revert to normal within 6 months and around 70% within 12 months without intervention and because the absolute event rate in the erythrocytic stratum in large unselected cohorts is no higher than in the normal-hemoglobin reference [17,23]. Following the framework of the recent JAK2 wild-type erythrocytosis review [30], hematology referral on SGLT2i should be considered when at least one of the following is present: persistent erythrocytosis at two consecutive measurements; erythrocytosis accompanied by thrombocytosis, leukocytosis, palpable splenomegaly, microvascular symptoms, aquagenic pruritus, or unexplained thrombosis; disproportionate hemoglobin rise relative to the expected SGLT2i effect or supranormal hemoglobin in patients without smoking, COPD or low LVEF that would otherwise explain it; family history of PV or other MPN. In such patients, JAK2 V617F testing, serum erythropoietin measurement and bone marrow assessment per WHO or ICC criteria [11,12,37] are appropriate, with the diagnostic and therapeutic framework for JAK2-unmutated erythrocytosis as set out by expert groups [30,33,38]. For the second question, we did not identify a prospective study of SGLT2i specifically in MPN populations, and the present analysis explicitly excluded MPN patients. Two inferences are reasonable on current evidence. Because erythrocytosis in this non-MPN HF cohort is transient and not associated with thrombotic events, and because large diabetes and HF cohorts show no excess thrombotic risk on SGLT2i [17,22,23], an SGLT2i-attributable component of hemoglobin rise in an MPN patient is unlikely to drive incremental thrombotic risk above that already present from the MPN itself, provided conventional MPN risk management (hematocrit target <45%, aspirin or anticoagulation as indicated, cytoreduction when warranted [32,39]) is maintained. Also, the predictor profile we identified for sustained erythrocytosis (male sex, smoking, univariably lower LVEF, higher baseline hemoglobin) is distinct from the clinical phenotype of incident PV [39,40], and an MPN-related component should remain readily distinguishable on clinical and laboratory grounds in shared-care settings. A recent retrospective study that investigated clinical and laboratory predictors of PV compared to SGLT2i-associated erythrocytosis identified high platelet count (>272 × 10^9^/L) and high platelet-to-lymphocyte ratio (>123) as the laboratory variables with the highest discriminatory accuracy, together with palpable splenomegaly, leukocytosis and elevated LDH supporting a PV diagnosis, whereas higher serum erythropoietin levels and a history of prior thrombosis supported SGLT2i-associated polycythemia [41]. For the third question, surveillance should give at least equal weight to the downward tail of the hemoglobin response—failure to correct, or new development of, anemia—rather than concentrating on the upward (erythrocytosis) tail. Hemoglobin that remains low or falls during the first 6–12 months on SGLT2i below anemia thresholds should prompt a structured anemia work-up, including iron studies (ferritin and transferrin saturation), renal function and a search for occult blood loss, as well as reassessment of adherence and of competing causes of decompensation. In persisting anemia patients, further bone marrow assessment may be warranted in search of clonal myeloid disorder.

### Limitations

This is a single-center observational registry without a non-SGLT2i comparator, so causal attribution of hemoglobin trajectories to the drug, as well as the observed clinical and prognostic associations, is not possible. No systematic iron studies, reticulocyte counts or erythropoietin measurements were performed. Patients with known MPN were excluded from the current study and implications of our findings for MPN populations are therefore inferential rather than direct. The relatively high proportion of missing follow-up hemoglobin measurements (25.5% at 6 months and 37.0% at 12 months) is an important limitation; although patients with and without a follow-up measurement did not differ in baseline hemoglobin or hemoglobin-category distribution, those lost to follow-up had more advanced heart failure, so the late landmark and transition estimates are likely biased toward the null. The MACE and especially the thrombosis endpoints had relatively few events, particularly at 12 months, which limits power for those landmark analyses and precluded a formal sex by erythrocytosis interaction test. Prediction models were derived by backwards regression analysis, which can overfit, particularly for the low-event erythrocytosis outcome. To mitigate this, the 6-month models were subjected to bootstrap internal validation [42] and penalized (LASSO and Firth) [43] re-estimation, which confirmed baseline hemoglobin as the robust dominant predictor while indicating that the secondary predictors of erythrocytosis are exploratory. Prognostic models were adjusted for clinically meaningful variables; however, anemic patients had features of more advanced disease, greater comorbidity burden, and residual confounding by disease severity is therefore likely, and anemia may in part be a marker of intrinsically sicker patients rather than a fully independent causal risk factor. Most importantly, this is a single-center registry from one Croatian tertiary center; its locally specific baseline hematologic profile, ethnic composition, referral pathways and follow-up practices may not be representative of other heart-failure populations, and our findings, particularly the proposed hemoglobin-monitoring recommendations, should be regarded as hypothesis-generating and require external, preferably multicenter and multi-ethnic, validation before broad generalization.

## 5. Conclusions

In HF patients receiving SGLT2i therapy, anemia, present at baseline or developing on treatment, identifies a high-risk subgroup with up to 2.5-fold increased all-cause mortality and 3-fold increased 6-month MACE risk, independently of age, sex, LVEF, eGFR and NT-proBNP. Among baseline-normal patients, downward transition to anemia is at least as common as upward transition to erythrocytosis, and is concentrated in vasculopathic, multimorbid and non-adherent patients. Erythrocytosis is uncommon, transient, and was not associated with mortality, MACE or thrombosis in this cohort; however, the study was underpowered for the thrombosis endpoint and no inference about the thrombotic safety of SGLT2i-associated erythrocytosis can be drawn, so these data should be read as the absence of a detectable signal rather than as evidence of safety. Routine monitoring of hemoglobin for anemia seems to be of higher clinical importance compared to erythrocytosis.

## Figures and Tables

**Table 1:** Baseline characteristics by baseline hemoglobin status. Continuous values: median (interquartile range). Categorical values: n (%). *p*-values: Kruskal–Wallis test for continuous variables; chi-square or Fisher’s exact test for categorical variables. Statistically significant *p*-values (*p* < 0.05). Abbreviations: BMI = body mass index; BP = blood pressure; bpm = beats per minute; HFrEF/HFmrEF/HFpEF = heart failure with reduced/mildly reduced/preserved ejection fraction; LVEF = left ventricular ejection fraction; NT-proBNP = N-terminal pro-B-type natriuretic peptide; NYHA = New York Heart Association; CAD = coronary artery disease; PAD = peripheral arterial disease; COPD = chronic obstructive pulmonary disease; OSA = obstructive sleep apnea; MCV = mean corpuscular volume; RDW = red cell distribution width; eGFR = estimated glomerular filtration rate; CRP = C-reactive protein; HbA1c = glycated hemoglobin.

Variable	Overall (N = 1244)	Anemia (n = 294)	Normal (n = 884)	Erythrocytosis (n = 66)	*p*
Age, years	69 (61–76)	75 (67–80)	68 (60–75)	65 (55–71)	<0.001
Female sex, n (%)	420 (33.8)	114 (38.8)	296 (33.5)	10 (15.2)	0.001
BMI, kg/m^2^	29.0 (25.7–32.6)	27.8 (25.1–31.9)	29.3 (25.8–32.9)	29.2 (25.8–31.5)	0.050
Systolic BP, mmHg	133 (120–150)	130 (115–145)	135 (121–150)	135 (119–150)	0.003
Heart rate, bpm	83 (71–100)	85 (70–100)	83 (70–100)	90 (77–105)	0.100
Dapagliflozin, n (%)	623 (50.4)	166 (56.5)	421 (47.9)	36 (55.4)	0.029
HFrEF/HFmrEF/HFpEF, %	58/15/26	53/19/29	59/14/26	68/12/20	0.109
LVEF, %	40 (30–50)	40 (30–50)	40 (30–50)	35 (25–45)	0.001
NT-proBNP, pg/mL	2419 (988–6173)	4548 (1853–11,746)	1957 (887–4803)	2152 (866–5789)	<0.001
NYHA class	2 (2–3)	3 (2–3)	2 (2–3)	2 (2–3)	<0.001
Hypertension, n (%)	1057 (85.0)	265 (90.1)	743 (84.0)	49 (74.2)	0.002
Diabetes mellitus, n (%)	514 (41.4)	158 (54.1)	334 (37.8)	22 (33.3)	<0.001
CAD, n (%)	653 (54.2)	150 (53.8)	475 (55.2)	28 (43.8)	0.206
PAD, n (%)	202 (16.3)	85 (28.9)	110 (12.5)	7 (10.6)	<0.001
COPD/asthma/OSA, n (%)	155 (12.5)	48 (16.3)	100 (11.3)	7 (10.6)	0.070
Current smoker, n (%)	420 (33.8)	78 (26.5)	308 (34.8)	34 (51.5)	<0.001
Hemoglobin, g/L	138 (126–149)	114 (104–121)	141 (134–150)	169 (165–175)	<0.001
Hematocrit, L/L	0.42 (0.38–0.45)	0.35 (0.32–0.37)	0.43 (0.40–0.46)	0.50 (0.49–0.52)	<0.001
MCV, fL	90.6 (87.3–94.0)	88.7 (83.4–93.7)	90.9 (88.0–93.8)	93.1 (91.4–96.1)	<0.001
RDW, %	14.1 (13.4–15.1)	15.3 (14.1–16.9)	13.9 (13.3–14.6)	13.6 (13.2–14.5)	<0.001
Platelets, ×10^9^/L	228 (186–274)	240 (185–296)	226 (186–267)	208 (188–238)	0.001
eGFR, mL/min/1.73 m^2^	66.6 (48.9–84.0)	53.3 (36.7–71.5)	69.6 (54.0–86.0)	73.0 (57.5–86.0)	<0.001
Albumin, g/L	40 (37–43)	37 (33–41)	41 (38–43)	39 (36–43)	<0.001
CRP, mg/L	5.6 (2.2–13.7)	7.8 (3.6–22.0)	4.8 (2.0–11.8)	4.3 (2.4–8.4)	<0.001
HbA1c, %	6.2 (5.8–6.8)	6.3 (5.9–7.0)	6.1 (5.7–6.7)	6.0 (5.6–6.7)	0.009

## Data Availability

The de-identified individual-participant data underlying this analysis are available from the corresponding author upon reasonable request, subject to institutional and ethics committee approval.

## Supplementary Materials

The following supporting information can be downloaded at https://www.mdpi.com/article/10.3390/jcm15145465/s1: Supplementary Table S1, univariable logistic regression for erythrocytosis (vs. normal hemoglobin) at baseline, 6 months and 12 months; Supplementary Table S2, univariable logistic regression for anemia (vs. normal hemoglobin) at baseline, 6 months and 12 months; Supplementary Table S3, detailed landmark Cox proportional hazards estimates for all-cause death and MACE at three landmark times (baseline, 6 months, 12 months); Supplementary Table S4, time-varying Cox proportional hazards model for all-cause death, with hemoglobin category modelled as a time-varying covariate across three exposure intervals per patient (baseline–6 months, 6–12 months, beyond 12 months); Supplementary Table S5, comparison of baseline characteristics between patients with and without an available 12-month hemoglobin measurement; Supplementary Table S6, comparison of patients with persistent, resolved and newly developed anemia; Supplementary Figure S1, row-percentage transition heatmaps for the three pairwise hemoglobin-category transitions among patients with paired measurements (baseline to 6 months n = 927, 6 to 12 months n = 749, baseline to 12 months n = 784); Supplementary Figure S2, hemoglobin category dynamics by SGLT2i agent (dapagliflozin vs. empagliflozin), real registry data.
