# Supplementary material for: Hemoglobin Trajectories on SGLT2 Inhibitor Therapy in Heart Failure: Anemia Marks Adverse Prognosis While Erythrocytosis Is Transient and Not Associated with Adverse Outcomes
Source: J Clin Med. 2026 Jul 13;15(14):5465. doi: 10.3390/jcm15145465 (PMC13412759; doi:10.3390/jcm15145465)
Supplement: Supplementary file 1 [file jcm-15-05465-s001.zip › jcm-4383158-supplementary.pdf]

Supplementary Materials

Supplementary Table S1

Univariable logistic regression for erythrocytosis (vs. normal hemoglobin) at baseline, 6 months and 12 months. Values are OR (95% CI), p-value.

Candidate predictor	Baseline	6 months	12 months
Age (per +10 years)	0.75 (0.61–0.91), **p=0.004**	0.85 (0.69–1.05), p=0.124	0.90 (0.70–1.16), p=0.428
Female sex	0.36 (0.18–0.71), **p=0.003**	0.41 (0.22–0.76), **p=0.005**	0.30 (0.13–0.73), **p=0.008**
BMI (per +1 kg/m²)	0.97 (0.93–1.02), p=0.270	1.01 (0.97–1.05), p=0.713	1.01 (0.95–1.06), p=0.795
Current smoking	1.98 (1.20–3.27), **p=0.008**	1.78 (1.10–2.88), **p=0.020**	1.25 (0.68–2.30), p=0.476
Diabetes mellitus	0.82 (0.49–1.40), p=0.473	0.70 (0.42–1.17), p=0.169	0.98 (0.52–1.81), p=0.937
Hypertension	0.55 (0.31–0.98), **p=0.044**	1.02 (0.52–1.99), p=0.964	0.80 (0.36–1.78), p=0.588
Dyslipidemia	1.09 (0.62–1.91), p=0.764	0.92 (0.54–1.56), p=0.755	1.26 (0.61–2.60), p=0.533
Atrial fibrillation	1.18 (0.72–1.96), p=0.507	1.19 (0.73–1.92), p=0.485	1.05 (0.57–1.92), p=0.882
Coronary artery disease	0.64 (0.38–1.06), p=0.082	0.83 (0.51–1.34), p=0.446	1.12 (0.61–2.05), p=0.709
Peripheral arterial disease	0.83 (0.37–1.86), p=0.650	1.16 (0.59–2.28), p=0.672	1.28 (0.58–2.84), p=0.543
Chronic kidney disease (eGFR<60)	0.74 (0.42–1.29), p=0.288	0.79 (0.47–1.34), p=0.381	1.45 (0.79–2.67), p=0.236
COPD or asthma	0.92 (0.41–2.08), p=0.849	1.98 (1.05–3.73), **p=0.034**	1.28 (0.52–3.15), p=0.586
Prior stroke	0.60 (0.21–1.70), p=0.338	1.20 (0.53–2.76), p=0.660	1.38 (0.52–3.64), p=0.520
HF phenotype (1=pEF,2=mrEF,3=rEF)	1.25 (0.91–1.70), p=0.167	1.03 (0.78–1.36), p=0.850	1.22 (0.84–1.78), p=0.295
LVEF (per +10%)	0.73 (0.59–0.91), **p=0.004**	0.89 (0.73–1.09), p=0.270	0.78 (0.60–1.01), p=0.064
NT-proBNP (per +1000 pg/mL)	1.02 (0.98–1.07), p=0.306	0.99 (0.95–1.04), p=0.672	1.00 (0.95–1.05), p=0.969
eGFR (per +10 mL/min/1.73m²)	1.04 (0.92–1.17), p=0.554	1.05 (0.94–1.17), p=0.433	0.91 (0.79–1.04), p=0.149
Serum sodium (per +1 mmol/L)	1.01 (0.95–1.07), p=0.811	0.97 (0.93–1.01), p=0.104	0.96 (0.86–1.06), p=0.397
Serum potassium (per +1 mmol/L)	1.01 (0.95–1.07), p=0.862	1.34 (0.81–2.22), p=0.249	1.34 (0.72–2.50), p=0.360
Serum albumin (per +5 g/L)	0.81 (0.58–1.12), p=0.203	0.69 (0.51–0.95), **p=0.023**	0.91 (0.59–1.39), p=0.651
C-reactive protein (per +10 mg/L)	0.88 (0.74–1.05), p=0.169	0.98 (0.89–1.09), p=0.766	0.97 (0.86–1.10), p=0.655
RDW (per +1%)	0.93 (0.74–1.17), p=0.549	0.82 (0.66–1.03), p=0.089	1.09 (0.91–1.30), p=0.346
Heart rate (per +10 bpm)	1.12 (1.01–1.25), **p=0.028**	1.09 (0.98–1.21), p=0.103	0.98 (0.85–1.12), p=0.725
MMAS-8 moderate (vs high)	-	0.55 (0.28–1.07), p=0.078	0.91 (0.45–1.84), p=0.802
MMAS-8 low (vs high)	-	1.05 (0.55–2.03), p=0.874	0.80 (0.33–1.94), p=0.622
Empagliflozin (vs dapagliflozin)	0.77 (0.47–1.27), p=0.307	0.94 (0.58–1.51), p=0.787	0.84 (0.46–1.55), p=0.583
Baseline hemoglobin (per +10 g/L)	-	2.37 (1.93–2.91), **p<0.001**	1.80 (1.45–2.24), **p<0.001**

Abbreviations: OR = odds ratio; CI = confidence interval; BMI = body mass index; COPD = chronic obstructive pulmonary disease; HF = heart failure; HFpEF = heart failure with preserved ejection fraction; HFmrEF = heart failure with mildly reduced ejection fraction; HFrEF = heart failure with reduced ejection fraction; LVEF = left ventricular ejection fraction; NT-proBNP = N-terminal pro–B-type natriuretic peptide; eGFR = estimated glomerular filtration rate; RDW = red cell distribution width; bpm = beats per minute; MMAS-8 = 8-item Morisky Medication Adherence Scale. Statistically significant associations (p<0.05) are shown in bold.

Supplementary Table S2

Univariable logistic regression for anemia (vs. normal hemoglobin) at baseline, 6 months and 12 months. Values are OR (95% CI), p-value.

Candidate predictor	Baseline	6 months	12 months
Age (per +10 years)	1.69 (1.47–1.94), **p<0.001**	1.72 (1.44–2.04), **p<0.001**	2.08 (1.69–2.58), **p<0.001**
Female sex	1.26 (0.96–1.66), p=0.097	0.92 (0.65–1.29), p=0.629	1.34 (0.92–1.96), p=0.130
BMI (per +1 kg/m²)	0.97 (0.95–1.00), **p=0.036**	0.96 (0.93–0.99), **p=0.018**	0.98 (0.94–1.01), p=0.169
Current smoking	0.67 (0.50–0.90), **p=0.008**	0.84 (0.59–1.18), p=0.316	0.67 (0.45–1.01), p=0.059
Diabetes mellitus	1.91 (1.47–2.50), **p<0.001**	2.05 (1.48–2.84), **p<0.001**	2.67 (1.82–3.90), **p<0.001**
Hypertension	1.75 (1.14–2.67), **p=0.010**	1.61 (0.95–2.70), p=0.074	1.77 (0.94–3.34), p=0.077
Dyslipidemia	0.83 (0.63–1.10), p=0.197	0.96 (0.67–1.37), p=0.832	0.86 (0.57–1.29), p=0.461
Atrial fibrillation	1.28 (0.99–1.67), p=0.064	1.54 (1.11–2.12), **p=0.009**	1.28 (0.89–1.86), p=0.186
Coronary artery disease	0.90 (0.69–1.18), p=0.446	1.02 (0.74–1.42), p=0.881	1.26 (0.87–1.83), p=0.223
Peripheral arterial disease	2.84 (2.06–3.92), **p<0.001**	3.04 (2.08–4.44), **p<0.001**	2.66 (1.73–4.09), **p<0.001**
Chronic kidney disease (eGFR<60)	2.72 (2.08–3.57), **p<0.001**	2.49 (1.79–3.45), **p<0.001**	2.92 (2.00–4.27), **p<0.001**
COPD or asthma	1.52 (1.05–2.21), p=0.028	1.64 (1.04–2.60), **p=0.033**	1.45 (0.84–2.49), p=0.178
Prior stroke	1.06 (0.68–1.65), p=0.790	1.74 (1.05–2.88), **p=0.032**	1.59 (0.88–2.85), p=0.123
HF phenotype (1=pEF,2=mrEF,3=rEF)	0.89 (0.76–1.03), p=0.120	0.88 (0.73–1.06), p=0.168	0.77 (0.63–0.95), **p=0.014**
LVEF (per +10%)	1.11 (1.00–1.23), p=0.059	1.08 (0.94–1.23), p=0.269	1.18 (1.02–1.37), **p=0.027**
NT-proBNP (per +1000 pg/mL)	1.09 (1.07–1.11), **p<0.001**	1.06 (1.04–1.08), **p<0.001**	1.06 (1.03–1.09), **p<0.001**
eGFR (per +10 mL/min/1.73m²)	0.74 (0.70–0.79), **p<0.001**	0.79 (0.73–0.85), **p<0.001**	0.72 (0.66–0.79), **p<0.001**
Serum sodium (per +1 mmol/L)	0.99 (0.97–1.02), p=0.549	0.94 (0.90–0.99), **p=0.030**	0.93 (0.88–0.99), **p=0.029**
Serum potassium (per +1 mmol/L)	1.01 (0.97–1.05), p=0.804	1.83 (1.33–2.52), **p<0.001**	2.33 (1.58–3.44), **p<0.001**
Serum albumin (per +5 g/L)	0.44 (0.36–0.53), **p<0.001**	0.51 (0.40–0.63), **p<0.001**	0.56 (0.43–0.73), **p<0.001**
C-reactive protein (per +10 mg/L)	1.09 (1.05–1.13), **p<0.001**	1.09 (1.04–1.15), **p<0.001**	1.01 (0.95–1.07), p=0.739
RDW (per +1%)	1.98 (1.78–2.21), **p<0.001**	1.61 (1.45–1.80), **p<0.001**	1.42 (1.28–1.59), **p<0.001**
Heart rate (per +10 bpm)	0.99 (0.93–1.05), p=0.752	1.01 (0.94–1.08), p=0.789	1.01 (0.92–1.09), p=0.887
MMAS-8 moderate (vs high)	-	1.09 (0.75–1.57), p=0.659	1.19 (0.79–1.79), p=0.413
MMAS-8 low (vs high)	-	2.04 (1.39–2.98), **p<0.001**	1.88 (1.21–2.92), **p=0.005**
Empagliflozin (vs dapagliflozin)	0.71 (0.55–0.93), **p=0.012**	0.79 (0.57–1.09), p=0.158	1.04 (0.72–1.50), p=0.853
Baseline hemoglobin (per +10 g/L)	-	0.41 (0.35–0.47), **p<0.001**	0.50 (0.44–0.57), **p<0.001**

Abbreviations: OR = odds ratio; CI = confidence interval; BMI = body mass index; COPD = chronic obstructive pulmonary disease; HF = heart failure; HFpEF = heart failure with preserved ejection fraction; HFmrEF = heart failure with mildly reduced ejection fraction; HFrEF = heart failure with reduced ejection fraction; LVEF = left ventricular ejection fraction; NT-proBNP = N-terminal pro–B-type natriuretic peptide; eGFR = estimated glomerular filtration rate; RDW = red cell distribution width; bpm = beats per minute; MMAS-8 = 8-item Morisky Medication Adherence Scale. Statistically significant associations (p<0.05) are shown in bold.

Supplementary Table S3

Detailed landmark Cox proportional hazards estimates for all-cause death and MACE at three landmark times (baseline, 6 months, 12 months). All models adjusted for age, sex, LVEF, eGFR and NT-proBNP. Reference: normal hemoglobin.

Endpoint and landmark	Anemia, aHR (95% CI), p	Erythrocytosis, aHR (95% CI), p
All-cause death — Baseline landmark (deaths=140)	2.23 (1.54–3.22), **p<0.001**	1.27 (0.59–2.74), ns
All-cause death — 6-month landmark (deaths=60)	2.45 (1.45–4.16), **p=0.001**	0.74 (0.22–2.49), ns
All-cause death — 12-month landmark (deaths=28)	1.90 (0.89–4.07), p=0.097	0.76 (0.05–11.50), ns
MACE — Baseline landmark	1.08 (0.58–2.02), ns	—
MACE — 6-month landmark	3.07 (1.27–7.38), **p=0.012**	—
MACE — 12-month landmark	—	—

Abbreviations: aHR = adjusted hazard ratio; CI = confidence interval; MACE = major adverse cardiovascular events; LVEF = left ventricular ejection fraction; eGFR = estimated glomerular filtration rate; NT-proBNP = N-terminal pro–B-type natriuretic peptide; ns = non-significant. Statistically significant associations (p<0.05) are shown in bold.

Supplementary Table S4

Time-varying Cox proportional hazards model for all-cause death, with hemoglobin category modelled as a time-varying covariate across three exposure intervals per patient (baseline–6 months, 6–12 months, beyond 12 months). Adjusted for age, sex, LVEF, eGFR and NT-proBNP. Reference: normal hemoglobin.

Hemoglobin category (time-varying)	Hazard Ratio	95% CI	p-value
Normal hemoglobin (reference)	1.00 (ref.)	—	—
Anemia	2.55	1.80–3.62	<0.001
Erythrocytosis	1.20	0.52–2.72	ns

Abbreviations: CI = confidence interval; LVEF = left ventricular ejection fraction; eGFR = estimated glomerular filtration rate; NT-proBNP = N-terminal pro–B-type natriuretic peptide; ref. = reference category; ns = non-significant.

Supplementary Table S5

Comparison of baseline characteristics between patients with and without an available 12-month hemoglobin measurement.

Characteristic	With 12-mo Hgb (n=784)	Without 12-mo Hgb (n=460)	p
Age, years	69 (61–75)	71 (62–78)	**0.001**
BMI, kg/m²	29.1 (25.7–33.0)	28.7 (25.6–31.9)	0.193
Heart rate, bpm	82 (70–99)	88 (72–104)	**0.015**
LVEF, %	40 (30–50)	40 (30–50)	0.716
NT-proBNP, pg/mL	2064 (976–5246)	3040 (1138–7855)	**<0.001**
eGFR, mL/min/1.73m²	67 (49–86)	66 (50–81)	0.161
Serum sodium, mmol/L	139 (138–141)	139 (136–140)	**<0.001**
Serum potassium, mmol/L	4.3 (4.0–4.6)	4.3 (4.0–4.6)	0.572
Serum albumin, g/L	41 (38–43)	39 (35–43)	**<0.001**
CRP, mg/L	5.3 (2.1–11.9)	6.2 (2.5–15.4)	0.081
RDW, %	14.1 (13.4–15.0)	14.0 (13.3–15.3)	0.826
Baseline hemoglobin, g/L	138 (126–148)	139 (124–150)	0.559

Characteristic	With 12-mo Hgb (n=784)	Without 12-mo Hgb (n=460)	p
Female sex	260 (33%)	160 (35%)	0.539
Current smoking	274 (35%)	147 (32%)	0.296
Diabetes mellitus	328 (42%)	187 (41%)	0.709
Hypertension	679 (87%)	380 (83%)	0.072
Dyslipidemia	580 (74%)	295 (64%)	**<0.001**
Atrial fibrillation	342 (44%)	213 (46%)	0.338
Coronary artery disease	414 (53%)	241 (52%)	0.924
Peripheral arterial disease	135 (17%)	67 (15%)	0.228
Chronic kidney disease (eGFR<60)	297 (38%)	187 (41%)	0.317
COPD or asthma	89 (11%)	66 (14%)	0.118
Prior stroke	71 (9%)	49 (11%)	0.350
HF phenotype: HFrEF	471 (60%)	255 (55%)	0.121
HF phenotype: HFmrEF	107 (14%)	85 (18%)	**0.022**
HF phenotype: HFpEF	208 (27%)	120 (26%)	0.884
MMAS-8 adherence: high	363 (46%)	48 (10%)	**<0.001**
MMAS-8 adherence: moderate	206 (26%)	34 (7%)	**<0.001**
MMAS-8 adherence: low	139 (18%)	49 (11%)	**<0.001**
MMAS-8 adherence: not taking	49 (6%)	21 (5%)	0.217
Empagliflozin (vs dapagliflozin)	374 (48%)	241 (52%)	0.101

Continuous variables are median (IQR), compared with the Mann–Whitney test; categorical variables are n (%), compared with the chi-square or Fisher's exact test. Statistically significant results (p<0.05) are shown in bold. Abbreviations: LVEF = left ventricular ejection fraction; NT-proBNP = N-terminal pro–B-type natriuretic peptide; eGFR = estimated glomerular filtration rate; RDW = red cell distribution width; PAD = peripheral arterial disease; COPD = chronic obstructive pulmonary disease; HFrEF/HFmrEF/HFpEF = heart failure with reduced/mildly reduced/preserved ejection fraction; MMAS-8 = 8-item Morisky Medication Adherence Scale.

Supplementary Table S6

Comparison of patients with persistent, resolved and newly developed anemia (stable non-anemic patients shown for reference).

Characteristic	Persistent anemia (n=115)	Resolved anemia (n=110)	New-onset anemia (n=70)	Stable non-anemic (n=669)	p*
Age, years	75 (69–82)	72 (65–79)	73 (67–78)	67 (59–74)	**0.011**
BMI, kg/m²	28.0 (24.5–32.6)	27.7 (25.8–31.5)	27.7 (24.7–31.9)	29.3 (26.1–33.1)	0.947
Heart rate, bpm	82 (68–96)	84 (70–100)	90 (77–103)	81 (70–99)	**0.044**
LVEF, %	40 (35–50)	40 (30–50)	40 (30–52)	40 (30–48)	0.599
NT-proBNP, pg/mL	4507 (2055–8944)	4126 (1295–11470)	3950 (1567–8661)	1765 (831–4166)	0.970
eGFR, mL/min/1.73m²	51 (36–67)	61 (41–80)	57 (43–75)	71 (55–87)	**0.032**
Serum sodium, mmol/L	139 (137–141)	139 (137–140)	138 (136–139)	139 (138–141)	**0.032**
Serum potassium, mmol/L	4.4 (4.1–4.8)	4.4 (4.1–4.6)	4.3 (4.0–4.8)	4.3 (4.0–4.6)	0.390
Serum albumin, g/L	38 (33–41)	38 (34–41)	38 (35–42)	41 (39–43)	0.411
CRP, mg/L	7.6 (3.9–18.5)	7.9 (3.2–20.2)	8.6 (4.1–22.4)	4.2 (1.9–10.4)	0.874
RDW, %	15.4 (14.4–16.9)	15.0 (13.9–16.4)	14.6 (13.9–15.4)	13.9 (13.3–14.5)	**<0.001**
Baseline hemoglobin, g/L	111 (101–119)	118 (110–125)	136 (131–145)	143 (135–152)	**<0.001**
Female sex	43 (37%)	39 (35%)	27 (39%)	207 (31%)	0.907
Current smoking	30 (26%)	34 (31%)	25 (36%)	251 (38%)	0.375
Diabetes mellitus	68 (59%)	57 (52%)	41 (59%)	237 (35%)	0.492
Hypertension	104 (90%)	101 (92%)	65 (93%)	562 (84%)	0.840
Dyslipidemia	80 (70%)	75 (68%)	48 (69%)	489 (73%)	0.974
Atrial fibrillation	59 (51%)	47 (43%)	35 (50%)	284 (42%)	0.399

Characteristic	Persistent anemia (n=115)	Resolved anemia (n=110)	New-onset anemia (n=70)	Stable non-anemic (n=669)	p*
Coronary artery disease	61 (53%)	62 (56%)	40 (57%)	344 (51%)	0.826
Peripheral arterial disease	38 (33%)	26 (24%)	18 (26%)	82 (12%)	0.262
Chronic kidney disease (eGFR<60)	72 (63%)	54 (49%)	37 (53%)	210 (31%)	0.113
COPD or asthma	19 (17%)	20 (18%)	11 (16%)	68 (10%)	0.901
Prior stroke	15 (13%)	7 (6%)	10 (14%)	57 (9%)	0.156
HF phenotype: HFrEF	62 (54%)	61 (55%)	37 (53%)	411 (61%)	0.940
HF phenotype: HFmrEF	21 (18%)	16 (15%)	8 (11%)	90 (13%)	0.441
HF phenotype: HFpEF	32 (28%)	33 (30%)	25 (36%)	168 (25%)	0.522
MMAS-8 adherence: high	30 (26%)	39 (35%)	17 (24%)	311 (46%)	0.179
MMAS-8 adherence: moderate	36 (31%)	27 (25%)	13 (19%)	157 (23%)	0.148
MMAS-8 adherence: low	30 (26%)	29 (26%)	20 (29%)	94 (14%)	0.927
MMAS-8 adherence: not taking	11 (10%)	5 (5%)	11 (16%)	38 (6%)	**0.040**
Empagliflozin (vs dapagliflozin)	53 (46%)	45 (41%)	28 (40%)	342 (51%)	0.640

*Anemia status was defined at baseline and at the last available follow-up (the 12-month value, or the 6-month value where the 12-month value was missing). Continuous variables are median (IQR); categorical variables are n (%). *p compares the three anemia-trajectory groups (persistent, resolved, new-onset) by Kruskal–Wallis (continuous) or chi-square or Fisher's exact test (categorical); the stable non-anemic column is shown for reference only. Statistically significant results (p<0.05) are shown in bold. Abbreviations: LVEF = left ventricular ejection fraction; NT-proBNP = N-terminal pro–B-type natriuretic peptide; eGFR = estimated glomerular filtration rate; RDW = red cell distribution width; PAD = peripheral arterial disease; COPD = chronic obstructive pulmonary disease; HFrEF/HFmrEF/HFpEF = heart failure with reduced/mildly reduced/preserved ejection fraction; MMAS-8 = 8-item Morisky Medication Adherence Scale.*

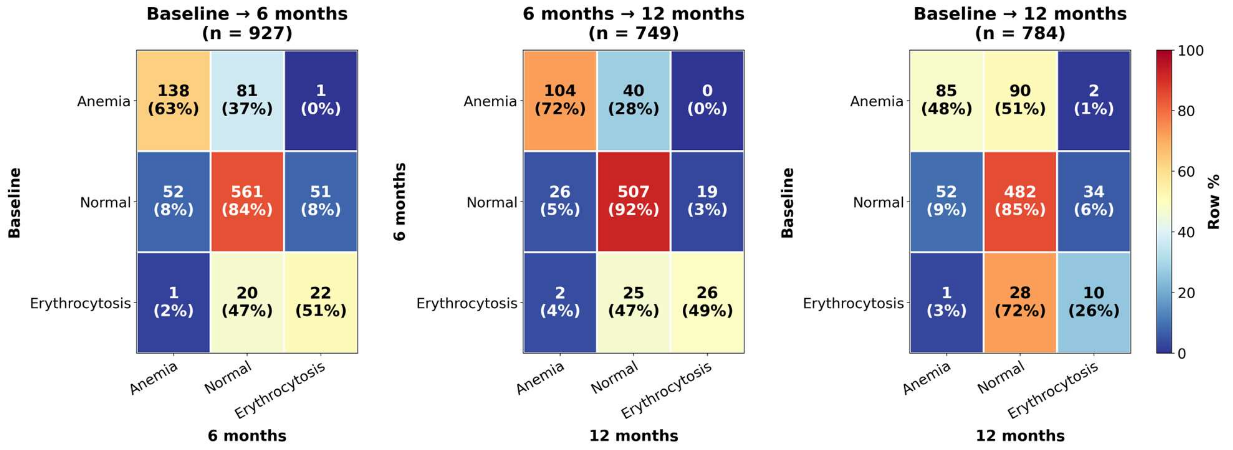

Supplementary Figure S1. Row-percentage transition heatmaps for the three pairwise hemoglobin-category transitions among patients with paired measurements (baseline to 6 months n=927, 6 to 12 months n=749, baseline to 12 months n=784). Each cell shows the count and the row percentage of patients moving from a given baseline category (rows) to a given follow-up category (columns); the color scale encodes the row percentage.

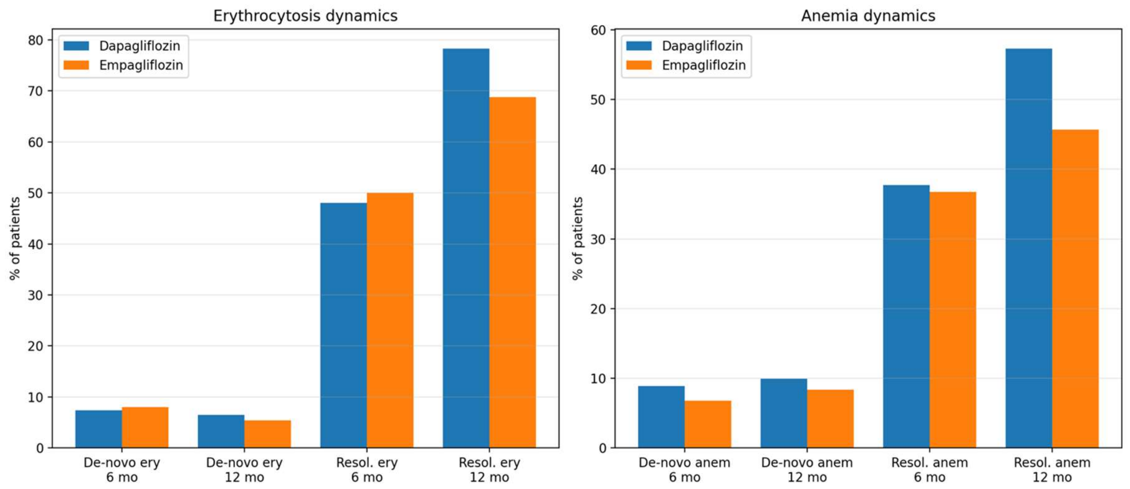

Supplementary Figure S2. Hemoglobin category dynamics by SGLT2i agent (dapagliflozin vs empagliflozin), real registry data. Left panel: de-novo erythrocytosis from baseline-normal at 6 and 12 months and resolution of baseline erythrocytosis. Right panel: de-novo anemia from baseline-normal and resolution of baseline anemia.
